# The Role of Platelet-Rich Plasma in the Management of Sensorineural Hearing Loss: Current Evidence and Emerging Trends

**DOI:** 10.7759/cureus.68646

**Published:** 2024-09-04

**Authors:** Chandra Veer Singh, Shraddha Jain

**Affiliations:** 1 Otolaryngology - Head and Neck Surgery, Jawaharlal Nehru Medical College, Datta Meghe Institute of Higher Education and Research, Wardha, IND

**Keywords:** clinical applications, hearing restoration, auditory tissue repair, regenerative therapy, platelet-rich plasma (prp), sensorineural hearing loss (snhl)

## Abstract

Sensorineural hearing loss (SNHL) is a common form of hearing impairment characterized by damage to the inner ear or auditory nerve, resulting in significant communication difficulties and reduced quality of life. Current treatment options, including hearing aids, cochlear implants, and corticosteroids, primarily focus on symptom management and do not address the underlying pathophysiological damage. Platelet-rich plasma (PRP), an autologous concentrate rich in platelets and growth factors, has emerged as a potential regenerative therapy due to its ability to promote tissue repair and cellular regeneration. This review provides a comprehensive overview of the role of PRP in the management of SNHL, examining the current evidence from preclinical and clinical studies. We discuss the mechanisms through which PRP may promote auditory tissue regeneration and repair, analyze its efficacy and safety profile, and explore innovative approaches and future directions in its application for SNHL. Despite promising preliminary findings, further research is needed to optimize PRP protocols, establish standardized treatment guidelines, and conduct large-scale randomized controlled trials to validate efficacy. This review aims to highlight the potential of PRP as a novel therapeutic strategy in treating SNHL and its possible integration into current clinical practices, offering new hope for patients with this debilitating condition.

## Introduction and background

Sensorineural hearing loss (SNHL) is a prevalent type of hearing impairment characterized by damage to the inner ear (cochlea) or the auditory nerve pathways leading to the brain [1]. It can result from various causes, including age-related degeneration (presbycusis), exposure to loud noises, ototoxic medications, genetic factors, and infections [2]. SNHL affects millions of people worldwide and can significantly impair communication abilities, leading to a decreased quality of life, social isolation, and increased risk of cognitive decline. Unlike conductive hearing loss, which involves problems in the outer or middle ear, SNHL typically involves irreversible damage to the sensory hair cells within the cochlea or the auditory nerve, making it more challenging to treat effectively [3].

The standard treatment options for SNHL focus primarily on managing symptoms rather than restoring hearing. These include hearing aids, cochlear implants, and, in some cases, corticosteroids or other medications to reduce inflammation or address autoimmune-related hearing loss [4]. While these interventions can improve hearing function and quality of life, they do not repair or regenerate the damaged auditory cells or nerve fibers. Hearing aids amplify sound but do not restore normal hearing, and their effectiveness can vary depending on the severity of the hearing loss and individual patient factors. Cochlear implants provide more direct stimulation to the auditory nerve, offering an alternative for those with profound hearing loss; however, they involve invasive surgery and do not fully replicate natural hearing [4]. Corticosteroids, typically used for sudden idiopathic hearing loss, offer limited benefits and are often only effective if administered early in the disease process. Thus, there is a significant unmet need for novel therapeutic approaches that can restore auditory function by targeting the underlying pathophysiology of SNHL [5].

Platelet-rich plasma (PRP) is an autologous biological product derived from a patient's blood, concentrated to contain a high level of platelets, growth factors, and cytokines. These components play a crucial role in wound healing and tissue regeneration, promoting cellular proliferation, differentiation, and angiogenesis [5]. In recent years, PRP has gained attention in various medical fields, including orthopedics, dermatology, and sports medicine, for its potential to enhance tissue repair and regeneration. The regenerative properties of PRP are thought to be particularly promising for treating conditions where traditional therapies have limited efficacy, such as SNHL [6]. The application of PRP in otology is based on the hypothesis that its growth factors could potentially promote the regeneration of cochlear hair cells and auditory nerve fibers, thus restoring some degree of hearing function [7].

This comprehensive review aims to explore PRP's emerging role in managing SNHL. It will provide an overview of the current evidence from basic science and clinical studies, discuss the mechanisms through which PRP may exert its effects on auditory tissues, and highlight this novel therapeutic approach's potential benefits and limitations. In addition, the review will examine future research directions and the possible integration of PRP into current treatment paradigms for SNHL, offering insights into its potential to transform the management of this challenging condition.

## Review

Understanding SNHL

Definition and Epidemiology of SNHL

SNHL is a form of hearing impairment resulting from damage to the inner ear (cochlea) or the auditory nerve [8]. This condition is defined by an air conduction threshold greater than 25 dB, with an air-bone gap of less than 10 dB in one or both ears. SNHL is a common health concern globally, affecting millions of individuals. In the United States alone, approximately 21 million people have some degree of hearing impairment, with around 1% classified as profoundly hearing impaired [9]. The incidence of hearing loss in neonates is about 1.1 per 1,000 births, although this rate can vary significantly by region, with some states reporting rates as low as 0.22 and others as high as 3.61 per 1,000. In India, the prevalence of unilateral SNHL (USNHL) is estimated to affect between 7.9% and 13.3% of the population, particularly among individuals aged 36 to 45 years [9]. Sudden SNHL (SSNHL), a specific type of SNHL characterized by its rapid onset, has an incidence rate ranging from five to 20 cases per 100,000 people, with most cases being unilateral. These statistics underscore the widespread nature of SNHL and emphasize the importance of effective screening and intervention strategies [10].

Pathophysiology of SNHL: Causes and Mechanisms

SNHL pathophysiology is complex and can be attributed to various factors. Genetic disorders are a major contributor, with approximately 70% of genetic hearing loss being nonsyndromic and 30% syndromic. Infections, particularly viral infections such as mumps and bacterial infections like meningitis, are also known causes of SNHL. In addition, prolonged exposure to loud noises can damage the delicate hair cells in the inner ear, resulting in permanent hearing loss [10]. Age-related hearing loss, or presbycusis, is another prevalent cause, especially among the elderly. Moreover, certain medications, particularly ototoxic drugs like aminoglycosides and cisplatin, can induce SNHL as a side effect. Understanding these causes and mechanisms is essential for developing targeted interventions and treatments for individuals with SNHL. Early diagnosis and management are crucial, as they can significantly improve patient outcomes, especially when the underlying causes are effectively addressed [11].

Impact of SNHL on the Quality of Life and Healthcare Burden

The impact of SNHL on an individual's quality of life can be substantial. Affected individuals often experience communication difficulties, which can lead to social isolation, anxiety, and depression. The inability to hear clearly can impede participation in social activities and strain personal relationships [12]. Patients with severe-to-profound SNHL may have difficulty locating the source of sounds and understanding speech, especially in noisy environments. The healthcare burden associated with SNHL is considerable. In the United States, approximately 4,000 infants are born with hearing impairment each year, underscoring the importance of early detection and intervention [13]. Worldwide, the disease burden caused by conditions like otitis media - a common precursor to SNHL - is significant, with an estimated 31 million episodes annually. Addressing the challenges of SNHL requires a comprehensive strategy that includes early detection, effective management, and rehabilitation. Emerging therapies, such as PRP treatment, offer promising prospects for managing certain types of SNHL by targeting the underlying cellular mechanisms responsible for hearing loss [14].

PRP: mechanisms and applications

**Figure 1:**
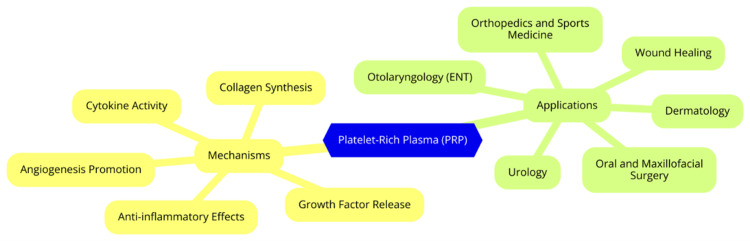
Mechanisms and applications of platelet-rich plasma (PRP) Image credit: Dr. Chandra Veer Singh

PRP, or autologous conditioned plasma, is a concentration of platelets and growth factors derived from a patient’s blood. The preparation process involves centrifugation to separate the plasma from red blood cells, resulting in a product with a platelet concentration typically three to five times higher than normal physiological levels [15]. PRP can be classified into several types based on the presence of leukocytes and fibrin content, including leukocyte-rich PRP (L-PRP), leukocyte-poor PRP (P-PRP), leukocyte platelet-rich fibrin (L-PRF), and pure platelet-rich fibrin (P-PRF) [16]. The therapeutic effects of PRP are primarily due to the growth factors and cytokines released from activated platelets [6]. When platelets are activated, they release various bioactive substances that play critical roles in healing and tissue regeneration, including platelet-derived growth factor (PDGF), transforming growth factor beta (TGF-β), fibroblast growth factor (FGF), vascular endothelial growth factor (VEGF), and insulin-like growth factors (IGF-1 and IGF-2). These growth factors enhance the healing process by promoting cell migration, proliferation, and differentiation, improving tissue regeneration and repair. The autologous nature of PRP reduces the risks of immune rejection and disease transmission, making it a safe option for a wide range of medical applications [17]. Historically, PRP has been used in various medical fields, including orthopedics for treating musculoskeletal injuries, dermatology for skin rejuvenation and hair loss conditions, dentistry for enhancing healing after oral and maxillofacial procedures, sports medicine for treating acute and chronic injuries, and plastic surgery for improving skin quality and accelerating healing following surgical interventions. As research continues, the applications of PRP may expand further, potentially transforming treatment protocols across multiple specialties [18]. The mechanisms and applications of PRP are illustrated in Figure 1.

PRP in otology: basic science and animal studies

Preclinical studies have offered valuable insights into the potential applications of PRP in managing SNHL. These investigations have focused on the effects of PRP on various components of the auditory system, including cochlear cells, hair cells, and nerve regeneration. By exploring these areas, researchers aim to uncover the mechanisms by which PRP may enhance hearing recovery [18]. Animal studies have demonstrated that PRP can have beneficial effects on cochlear cells. For example, research involving guinea pigs has shown that PRP treatment significantly increases the survival and proliferation of spiral ganglion neurons (SGNs) in the inner ear. This finding suggests that PRP may help preserve auditory nerve function in cases of SNHL, potentially leading to improved hearing outcomes. The ability of PRP to support the health and viability of cochlear cells is a critical aspect of its therapeutic potential [19]. In addition to its effects on cochlear cells, PRP has also been found to stimulate the regeneration of damaged hair cells, which is essential for converting sound vibrations into electrical signals that the brain can interpret. Studies using animal models indicate that PRP can promote the proliferation and differentiation of hair cell progenitors. This regenerative capacity highlights PRP's potential to restore hair cell function and improve hearing in patients suffering from SNHL. The ability to regenerate hair cells could represent a significant advancement in treating this condition [20]. Moreover, PRP has shown promise in promoting nerve regeneration within the inner ear. The growth factors present in PRP, such as platelet-derived growth factor (PDGF) and transforming growth factor-beta (TGF-β), play critical roles in nerve repair and regeneration. Animal studies have demonstrated that PRP can enhance the recovery of auditory nerve function following injury or degeneration. This aspect of PRP therapy underscores its potential to address hair cell damage and the broader challenges associated with auditory nerve impairment [21]. The beneficial effects of PRP in the inner ear can be attributed to several mechanisms. First, PRP exhibits anti-inflammatory properties, which can help reduce inflammation and oxidative stress - common contributors to SNHL. In addition, PRP stimulates angiogenesis or the formation of new blood vessels, thereby improving blood supply and nutrient delivery to inner ear tissues. Furthermore, PRP contains growth factors that activate signaling pathways in cell growth, proliferation, and differentiation, supporting cellular health and function. Finally, PRP can inhibit apoptosis, or programmed cell death, of hair cells and auditory neurons, preserving their functionality [22].

Clinical evidence of PRP in the management of SNHL

SNHL is a prevalent condition that can greatly affect an individual's quality of life. Traditional treatments, such as hearing aids, provide temporary relief by amplifying sound levels. Still, emerging therapies like PRP offer a more transformative approach by addressing the underlying cellular issues contributing to hearing loss [8]. PRP is a concentrated source of PRP protein derived from whole blood that is processed through centrifugation to remove red blood cells. It consists of two primary components: plasma, the liquid portion of blood composed mainly of water and proteins, and platelets, also known as thrombocytes, which play a crucial role in the body's healing processes. Platelets contain alpha and dense granules that release growth factors, instrumental in nerve regeneration and reducing inflammation [23]. PRP has shown promise in treating certain types of hearing loss, particularly cases where the hearing loss results from damage to the hair cells within the inner ear. Hair cell damage can occur due to aging, exposure to loud noises, or side effects from specific medications. PRP therapy involves injecting a concentrated dose of platelets derived from the patient’s blood into the inner ear to stimulate cell regeneration. Studies have indicated that PRP can effectively treat conductive and SNHL [24]. The outcomes of PRP treatment for SNHL can vary based on the severity and duration of the condition. Among patients with less than six months of hearing loss, 85.2% experienced complete recovery, while 14.8% showed partial recovery. For those with mild SNHL, 70.8% achieved complete recovery, and 29.2% had partial recovery. In cases of moderate SNHL, 25% had complete recovery, 50% had partial recovery, and 25% had no recovery. Patients with moderately severe hearing loss showed complete recovery in 66.7% of cases, partial recovery in 11.1%, and no recovery in 22.2%. In patients with severe SNHL, only 42.9% experienced complete recovery, 14.2% had partial recovery, and 42.9% showed no recovery [25]. While PRP treatment is generally considered safe, some patients may experience temporary side effects such as dizziness (41.4% of cases) and pain (61.4% of cases). These side effects are typically mild and resolve on their own. PRP therapy presents a promising approach for managing SNHL by targeting the underlying causes at a cellular level. Although the results are encouraging, further research is necessary to understand this treatment's long-term efficacy and safety fully. As with any medical intervention, it is essential to consult a qualified healthcare professional to determine the most appropriate course of action for each case [26].

Emerging trends and innovative approaches in PRP therapy for SNHL

While conventional PRP therapy has shown promising results in treating SNHL, researchers are exploring novel formulations and delivery methods to enhance its effectiveness. One area of research focuses on developing new techniques to obtain PRP formulations enriched in platelets and extraplatelet biomolecules. For instance, hydroxyethyl acrylamide (HEAA)-based hydrogels are being used to absorb water from the plasma, thereby concentrating the platelet and growth factor content [27]. This novel PRP (nPRP) formulation has demonstrated significantly higher levels of insulin-like growth factor-1 (IGF-1) and hepatocyte growth factor (HGF) compared to standard PRP (sPRP). In vitro studies have shown that nPRP improves cell viability in human dermal fibroblasts and primary chondrocytes [28]. Another innovative technique, temperature-controlled PRP (t-PRP), employs hypothermic conditions (4°C) during a two-step centrifugation process to prepare PRP without exogenous additives. T-PRP maintains a physiological pH, achieves a higher platelet concentration, and provides stable growth factor levels compared to contemporary PRP (c-PRP). T-PRP forms a natural fibrin scaffold upon activation that traps more platelets and growth factors, featuring a slow release and degradation rate [29]. Intratympanic injection of PRP directly into the inner ear has also shown promise in treating SNHL. This method allows targeted delivery of growth factors and other bioactive molecules to the affected area, potentially enhancing therapeutic effects. In addition, researchers are exploring microspheres as a delivery system for platelet-derived growth factors in wound healing applications. These biodegradable particles can encapsulate and slowly release growth factors, potentially improving their bioavailability and efficacy. Although this approach has not yet been studied in SNHL, it could be adapted for intratympanic delivery of PRP [30]. Combining PRP with other treatments, such as stem cells or additional growth factors, may further enhance its regenerative potential. For example, in animal models, PRP has been used alongside bone marrow-derived mesenchymal stem cells (MSCs) to treat SNHL. The growth factors in PRP can promote the survival and differentiation of the transplanted MSCs, potentially leading to improved outcomes [31]. Identifying biomarkers that predict an individual's response to PRP therapy could help optimize treatment strategies. Researchers are investigating various factors, such as specific growth factor receptors' presence and activation state on target cells, as potential predictive biomarkers. By tailoring PRP treatment based on individual patient characteristics, clinicians may improve outcomes and minimize non-response risk [31]. Emerging trends and innovative approaches in PRP therapy for SNHL are presented in Table 1.

**Table 1:** Emerging trends and innovative approaches in PRP therapy for sensorineural hearing loss PRP: platelet-rich plasma

Trend/approach	Description	Potential benefits	Current status
Novel PRP formulations [32]	Development of enhanced PRP preparations with optimized platelet concentrations or added growth factors.	It may improve efficacy by providing a more potent regenerative effect.	In the early stages of research, few clinical studies.
Advanced delivery methods [33]	Innovative delivery techniques include intratympanic injections, gel formulations, or biodegradable carriers.	Enhanced precision and sustained release of PRP to targeted auditory tissues.	Pilot studies and trials are underway.
Combination therapies [33]	Use of PRP in combination with other treatments like stem cells, gene therapy, or growth factors.	Potential synergistic effects that could enhance overall treatment outcomes.	Experimental phase; limited clinical evidence.
Biomarker identification [34]	Research into biomarkers predicting PRP treatment response, enabling personalized therapy approaches.	Improved patient selection and tailored treatment strategies.	Ongoing research; not yet widely implemented.
Regenerative medicine integration [35]	Combining PRP with other regenerative techniques such as tissue engineering or stem cell therapy.	It may offer more comprehensive solutions for cellular regeneration and functional recovery.	Emerging field with promising preliminary results.
Technological advancements [36]	Integration of PRP therapy with advanced imaging techniques or AI to optimize treatment planning.	Enhanced precision in targeting and monitoring treatment effects.	Early applications; research in progress.

Challenges and limitations in the use of PRP for SNHL

The application of PRP in managing SNHL presents several challenges and limitations that impede its efficacy and broader adoption. These challenges can be categorized into three main areas: technical difficulties in PRP preparation and standardization, variability in clinical protocols and outcomes, and limitations in the current body of evidence [27]. One of the primary challenges involves the technical aspects of PRP preparation. The lack of standardization in PRP protocols results in considerable variability in the concentration and composition of platelets and growth factors across different preparation methods. Various techniques, including open and closed systems, produce differing results, complicating the comparison of outcomes across studies. In addition, differences in processing methods, such as centrifugation speed, duration, and activation techniques, further contribute to this inconsistency [37]. For example, research has shown that platelet concentrations can vary widely depending on the method used, directly affecting PRP's therapeutic potential. Moreover, inadequate reporting of preparation methods in the literature exacerbates this issue, with only a small percentage of studies providing comprehensive details about their protocols. This lack of transparency hampers reproducibility and makes establishing standardized PRP preparation practices challenging [38].

Another significant challenge is the variability in clinical protocols and outcomes associated with PRP treatment for SNHL. The absence of standardized clinical protocols leads to inconsistencies in treatment administration, including variations in dosages, routes of administration, and follow-up procedures [39]. These discrepancies make it difficult to assess PRP's effectiveness in treating SNHL accurately. Furthermore, individual patient responses to PRP can vary widely due to factors such as overall health, the severity of hearing loss, and the duration of the condition before treatment. This variability can result in inconsistent clinical outcomes, complicating efforts to draw definitive conclusions about the efficacy of PRP for SNHL [38].

The limitations of the current evidence further exacerbate the challenges associated with PRP. A notable concern is the lack of large-scale RCTs that could provide robust data on the effectiveness of PRP for SNHL. Most existing studies are small, observational, or lack rigorous design, raising questions about the reliability and generalizability of their findings. The absence of well-designed clinical trials limits the ability to establish clear guidelines for using PRP in treating SNHL [40]. In addition, the heterogeneity in study designs, including differences in patient populations, outcome measures, and treatment protocols, complicates the interpretation of results. This diversity makes it difficult to conduct meta-analyses or systematic reviews that could provide more definitive insights into the effectiveness of PRP for SNHL [41]. The challenges and limitations in the use of PRP for SNHL are shown in Table 2.

**Table 2:** Challenges and limitations in the use of PRP for SNHL PRP: platelet-rich plasma, SNHL: sensorineural hearing loss

Challenge/limitations	Description	Impact
Variability in PRP preparation [42]	Differences in PRP preparation techniques, concentration of platelets, and presence of growth factors.	Affects consistency and effectiveness of treatment outcomes.
Lack of standardization [42]	No standardized protocols for PRP administration, including dosage, frequency, and delivery methods.	Hinders comparison of results across studies and clinical practices.
Limited large-scale clinical trials [43]	Insufficient number of high-quality, large-scale randomized controlled trials.	Limits the ability to draw definitive conclusions about efficacy and safety.
Inconsistent clinical results [43]	Variable efficacy was reported in different studies and patient populations.	Challenges in establishing clear guidelines for clinical use.
Technical and procedural challenges [44]	Issues related to the technical aspects of PRP injection and procedural complications.	Potential for adverse effects or suboptimal outcomes.
Understanding mechanisms of action [44]	Uncertainty about the precise mechanisms by which PRP affects auditory tissues.	Limits optimization of PRP protocols and targeted therapies.
Cost and accessibility [37]	High cost of PRP preparation and administration and limited availability of treatment centers.	This may restrict access to therapy and increase healthcare costs.

Future directions and research opportunities

Further basic science research is essential to fully comprehend how PRP influences SNHL treatment. Investigating the specific growth factors in PRP, such as platelet-derived growth factors (PDGF), transforming growth factor-beta (TGF-β), and vascular endothelial growth factor (VEGF), will shed light on how these components contribute to cellular proliferation, migration, and overall regeneration within the auditory system [37]. In addition, it is crucial to elucidate the precise cellular pathways activated by PRP that lead to hair cell regeneration and improved auditory function. Understanding these pathways can help optimize PRP formulation and application, enhancing its therapeutic efficacy [45]. Future clinical trials must be meticulously designed to ensure robust and reliable outcomes. RCTs should be prioritized to compare PRP with standard treatments such as corticosteroids or placebo. This design will help establish PRP's efficacy in a controlled environment. Patient selection is another critical factor; trials should encompass diverse demographics, focusing on age, duration of hearing loss, and underlying causes of SNHL [46]. Stratifying patients based on these variables will help identify which subgroups benefit most from PRP therapy. Furthermore, establishing clear and objective endpoints is essential. Audiometric improvements, quality of life assessments, and functional outcomes should be measured using standardized metrics to enhance result comparability across studies [47].

Integrating PRP into multimodal treatment strategies for SNHL could significantly improve therapeutic outcomes. One approach could involve combination therapies, where PRP is administered alongside other treatments, such as corticosteroids or cochlear implants. This strategy might offer synergistic benefits; PRP could be administered postoperatively to enhance recovery following cochlear implantation or alongside steroids to improve initial hearing recovery rates [27]. In addition, developing personalized treatment plans based on patient-specific factors, including SNHL severity and response to previous treatments, may optimize the benefits of PRP therapy. Longitudinal studies assessing the durability of PRP's effects in conjunction with other therapies will also be crucial for establishing comprehensive treatment protocols [27]. By addressing these areas, future research can significantly advance the understanding and application of PRP in managing SNHL, potentially leading to improved patient outcomes and quality of life [4]. Future directions and research opportunities for PRP in the management of SNHL are summarized in Table 3.

**Table 3:** Future directions and research opportunities for PRP in the management of SNHL PRP: platelet-rich plasma, SNHL: sensorineural hearing loss

Research area	Description	Potential impact
Optimization of PRP formulations [48]	Investigate different PRP preparation methods to standardize concentration and composition.	Improve consistency and efficacy of PRP treatments across studies and clinical applications.
Mechanistic studies in auditory tissue [38]	Conduct in-depth research to understand how PRP promotes regeneration in cochlear cells and neurons.	Enhance knowledge of PRP’s regenerative mechanisms specific to auditory tissues, guiding targeted therapies.
Development of novel delivery methods [49]	Explore new delivery methods (e.g., intratympanic injections, biodegradable scaffolds) for PRP.	Increase the precision and effectiveness of PRP delivery to the inner ear, reducing potential side effects.
Combination therapies with PRP [49]	Investigate the use of PRP in combination with other treatments (e.g., stem cells, growth factors).	Enhance overall treatment efficacy by leveraging synergistic effects, potentially leading to better outcomes.
Large-scale randomized controlled trials (RCTs) [50]	Conduct well-designed RCTs to assess the efficacy and safety of PRP in diverse patient populations.	Provide robust evidence to support or refute the use of PRP in clinical practice, influencing treatment guidelines.
Identification of biomarkers for treatment response [51]	Research biomarkers that predict which patients are most likely to benefit from PRP therapy.	Personalize treatment plans, improve outcomes and reduce unnecessary exposure to PRP for non-responders.

## Conclusions

PRP represents a promising frontier in managing SNHL, offering potential regenerative benefits beyond traditional therapies' capabilities. Current evidence, derived from preclinical and clinical studies, suggests that PRP may facilitate the repair and regeneration of damaged auditory cells and nerve fibers, potentially improving hearing function in individuals with SNHL. Despite these encouraging findings, the application of PRP in otology is still in its infancy, and several challenges remain, including the need for standardized protocols, a better understanding of the mechanisms of action, and more robust clinical trials to establish efficacy and safety. Nevertheless, PRP's unique ability to harness the body's natural healing processes offers a novel approach that could complement or even enhance existing treatments for SNHL. As research in this field progresses, there is hope that PRP could be integrated into a broader therapeutic strategy, paving the way for more effective and restorative treatments for individuals affected by SNHL.
